# Tunable wave coupling in periodically rotated Miura-ori tubes

**DOI:** 10.1098/rsta.2024.0006

**Published:** 2024-10-07

**Authors:** Sunao Tomita, Tomohiro Tachi

**Affiliations:** ^1^ Toyota Central R&D Labs Inc. 1-4-14 Koraku, Bunkyo-ku, Bunkyo-ku,Tokyo 112-0004, Japan; ^2^ Department of General Systems Studies, Graduate School of Arts and Sciences, The University of Tokyo, 3-8-1 Komaba, Meguro-Ku, Meguro-Ku,Tokyo, Japan

**Keywords:** origami, programmable materials, band gap

## Abstract

Origami folding structures are vital in shaping programmable mechanical material properties. Of particular note, tunable dynamical properties of elastic wave propagation in origami structures have been reported. Despite the promising features of origami metamaterials, the influence of the kinematics of tessellated origami structures on elastic wave propagation remain unexplored. This study proposes elastic metamaterials using connected Miura-ori tubes, the kinematics of which are coupled by folding and unfolding motions in a tubular axis; achieved by periodically connecting non-rotated and rotated Miura-ori tubes. The kinematics generate wave modes with localized deformations within the unit cell of the metamaterials, affecting the global elastic deformation of Miura-ori tubes via the coupling of wave modes. Dispersion analysis, using the generalized Bloch wave framework based on bar-and-hinge models, verifies the influence of kinematics in the connected tubes on elastic wave propagation. Furthermore, folding the connected tubes changes the coupling strength of wave modes between the kinematics and global elastic deformation of the tubes by breaking the ideal kinematics. The coupling of wave modescontributes to the formation of the band gaps and their tunability. These findings enable adaptive and *in situ* tunability of band structures to prohibit elastic waves in the desired frequency ranges.

This article is part of the theme issue ‘Origami/Kirigami-inspired structures: from fundamentals to applications’.

## 1. Introduction

Origami, the traditional art of paper folding, has gained much attention in science and technology. Origami facilitates the fabrication of diverse functional shapes from flat sheets, including curved surfaces [1,2], honeycombs [3,4] and cellular structures [5–7]. Further, the kinematics of origami facilitate the design of deformable structures across various scales, including non-invasive medical devices [8,9], flexible electronics [10,11], robots [12–14], foldable architectural structures [15–17] and space structures [18–20].

Cellular materials exhibiting unique mechanical properties, known as mechanical metamaterials, stem from the assembly of origami. An assembly of origami that does not retain its kinematics leads to stiff and lightweight cellular materials [21,22] or structures with high energy absorption [23,24]. On the other hand, an assembly of origami that retains its kinematics, such as the tubular composition of the Miura-ori pattern and Tachi-Miura polyhedra [25,26], leads to origami metamaterials with negative Poisson’s ratios [27,28], graded cellular structures [29], multistable cellular structures [30], programmable collapse [31], load-bearing capability [32], efficient energy absorption [33–35] and stiff yet flexible materials, such as zipper-coupled tubes [36] or mirror-coupled tubes [37].

The dynamic mechanical responses of origami metamaterials have also been studied. Origami metamaterials exhibit the behaviour of elastic waves, such as rarefaction waves formed in chains of origami structures [38,39]. Kresling patterns also form a wave converter between longitudinal and torsional waves using their folding motions [40]. The gradient geometries in Miura-ori realize phononic crystal lenses for Lamb-wave propagation [41]. Furthermore, the wave filtering of elastic waves, called band gaps (induced by origami patterns) have been observed in Miura-ori [42,43].

Moreover, the *in situ* geometrical transformation of origami structures provides further tunability of such wave behaviour. For instance, the frequency ranges of the band gaps are tuned using the origami method [44,45]. Additionally, chains composed of Kresling origami structures were investigated to realize tunable band gaps [46]. Moreover, distributed resonators with tunable resonant frequencies inherent in Kresling pattern bistability enable tunable band gaps for elastic waves in thin plates [47,48]. More recently, topological states in topological insulators have been transferred using Kresling origami [49]. Dispersion analyses were performed for Miura-ori [42,50] and egg-box pattern [42] to determine the tunable band structures of elastic waves in origami tesselation. In addition to elastic waves, the tunability of band gaps and wave localization for acoustic waves were investigated using a perforated Miura-ori [51]. Transformable acoustic lattices have also been developed using foldable cubic arrays [52] and Miura-ori with poles on the vertices [53,54] based on lattice transformation [55].

Despite the promising features of origami metamaterials, one of the remaining challenges is to produce band gaps in the low-frequency range. Existing methods rely on multiple materials for the panels [42], holes with the local resonators on the panels [45] or additional mass on the origami spring [48] to realize band gaps in the low-frequency ranges. The influence of the kinematics of tessellated origami structures on low-frequency elastic wave propagation remain unexplored. Therefore, we investigate origami structures for which the kinematics affect low-frequency elastic wave propagation. This, in particular, enables the simple realization of metamaterials purely based on the geometry of origami.

Specifically, this study utilizes non-rotated and rotated Miura-ori tubes coupled in a tubular axis [56] in which the folding and unfolding motions are coupled. This enables the elastic metamaterials that influence low-frequency elastic wave propagation as the coupled part acts as the resonator. Tailored wave propagation using connected Miura-ori tubes and tunability via folded connected tubes were analysed via a dispersion analysis of bar-and-hinge models [42]. The dispersion analysis clarified the influence of the kinematics of the connected Miura-ori tubes on elastic wave propagation. Furthermore, the complete low-frequency band gaps for all wave modes were explored using the coupling of wave modes caused by the kinematics of the interaction between non-rotated and rotated Miura-ori tubes to global elastic deformation. The findings of this study enable the design of band gaps in wider frequency ranges and their *in situ* adaptation via folding after the construction of elastic metamaterials.

## 2. Geometry and numerical modelling of Miura-ori tubes

### (a) Geometry and kinematics

**Figure 1.**
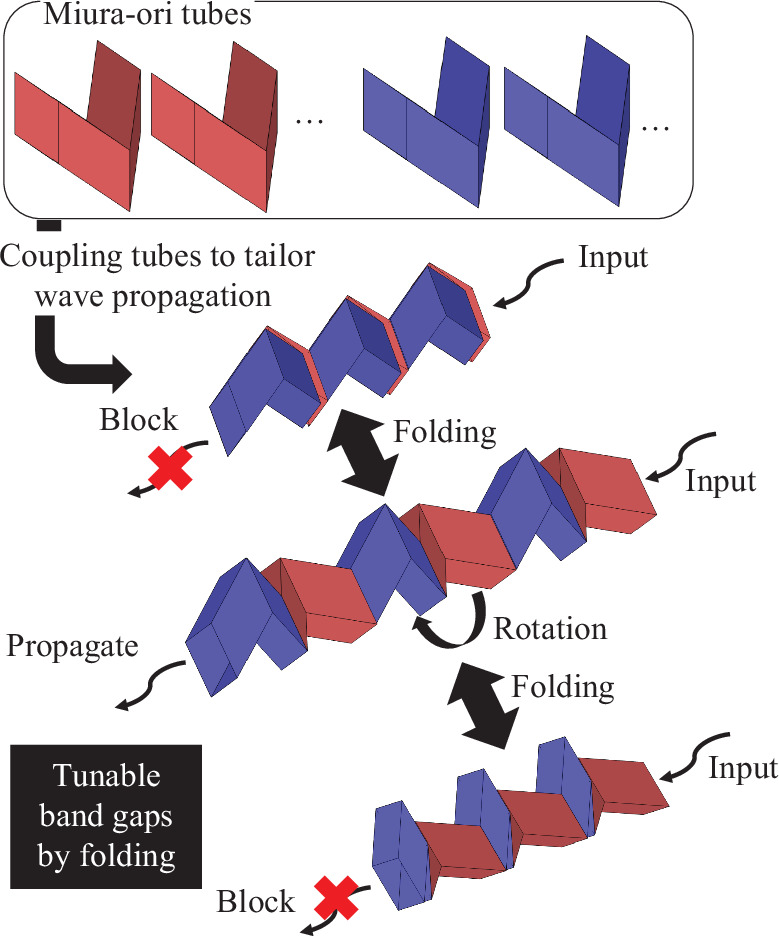
Schematic of the tunable waveguide by connecting Miura-ori tubes. Coupling Miura-ori tubes after rotation along the tubular axis provides various periodicity. Furthermore, the wave propagation can be tuned by changing the periodicity via transforming the structures induced by the folding of origami structures.

The Miura-ori tubes can be connected after rotating along the tubular axis [56]. Connection enables the design of various transformable structures, as shown in figure 1. This study aims to switch and tune wave propagation characteristics, such as band gaps in waveguides, by folding connected Miura-ori tubes. Therefore, elastic wave propagation within connected Miura-ori tubes was investigated in detail to change the band gaps and wave modes by folding.

**Figure 2.**
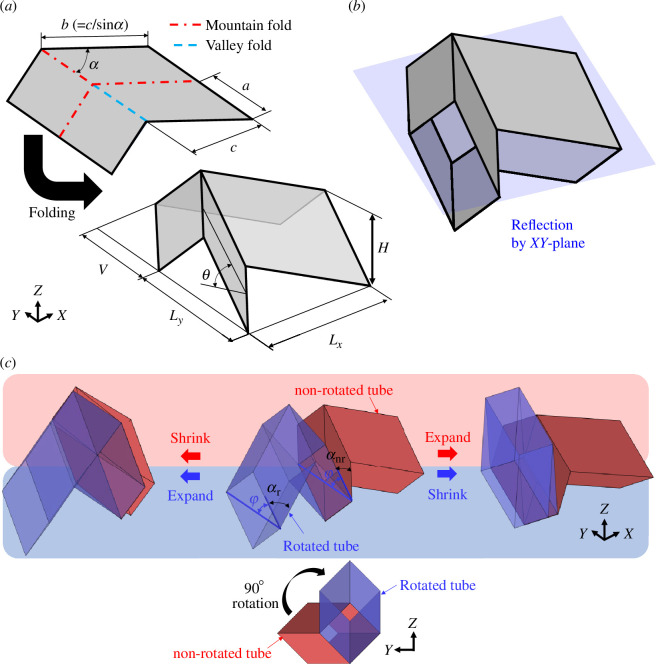
Geometry and motion of Miura-ori tubes connected between the non-rotated and rotated along the axis of the tubes. (*a*) The geometry of the Miura-ori unit. (*b*) Miura-ori tube formed by connecting the mirrored Miura-ori along the XY-plane. (*c*) Connection of the non-rotated and $90^{\circ }$-rotated Miura-ori tubes.


The geometry of the tunable waveguide is introduced by connecting the non-rotated and rotated Miura-ori tubes. The dimensions of the Miura-ori unit cell are presented in figure 2*a*. The length of Miura-ori a, b, internal angle of the parallelogram α, half dihedral angle θ∈[0,π/2] and dimensions of the unit cells are defined as follows:


(2.1) $$L_{x}=2b\frac{\cos\theta \tan\alpha }{\sqrt{1+\cos^{2}\theta \tan^{2}\alpha }},$$

(2.2) $$\begin{array}{ccc} & L_{y}=2a\sqrt{1-\sin^{2}\;\theta \;\sin^{2}\;\alpha }, & \end{array}$$

(2.3) $$\begin{array}{ccc} & H=2a\;\sin\;\theta \;\sin\;\alpha , & \end{array}$$

and


(2.4) $$\begin{array}{ccc} & V=b\frac{1}{\sqrt{1+\cos^{2}\;\theta \;\tan^{2}\;\alpha }}. & \end{array}$$

Miura-ori tubes were created by connecting the unit of Miura-ori to those mirrored along the XY-plane, as shown in figure 2*b*.

The Miura-ori tubes can be connected after the adjacent Miura-ori tube sections are rotated $90^{\circ }$ relative to each other. Compatibility after the connection between the non-rotated and rotated Miura-ori tubes is ensured using the angle φ in the cross-section of the interfaces, as shown in figure 2*c*. The range of angle φ is limited to $\left[90^{\circ }-\alpha_{r},\alpha_{nr}\right]$, where $\alpha_{r}$ and $\alpha_{nr}$ are the internal angles of the parallelograms of the rotated and non-rotated Miura-ori tubes, respectively. Using the shared angle φ, the lengths of Miura-ori tubes defined in equation (2.1) can be rewritten as


(2.5) $$\begin{array}{ccc} & L_{nr}=b_{nr}\frac{\sqrt{\sin^{2}\;\alpha_{nr}-\sin^{2}\;\varphi }}{\cos\;\varphi }, & \end{array}$$

and


(2.6) $$\begin{array}{ccc} & L_{r}=b_{r}\frac{\sqrt{\sin^{2}\;\varphi -\cos^{2}\;\alpha_{r}}}{\sin\;\varphi }. & \end{array}$$

The total lengths of the non-rotated and rotated Miura-ori tubes are


(2.7) $$\begin{array}{ccc} & L=L_{nr}+L_{r}. & \end{array}$$

**Figure 3.**
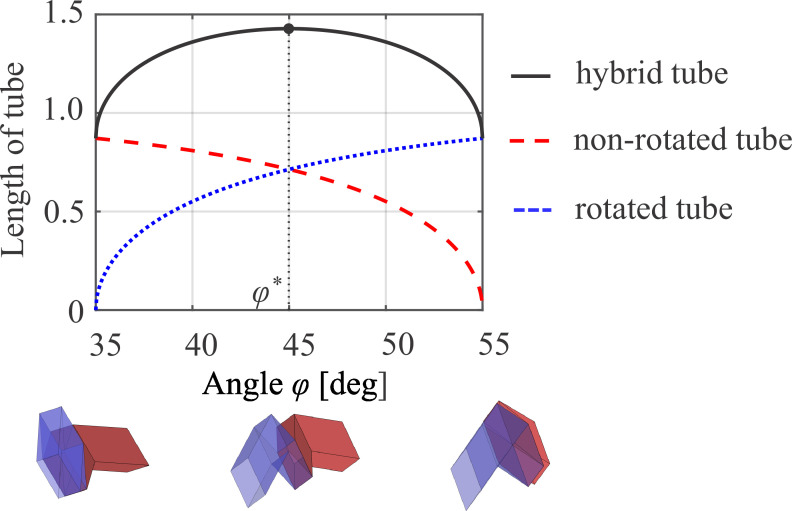
Length of hybrid tubes connected by the non-rotated and rotated Miura-ori tubes. By folding, the non-rotated and rotated tubes induce opposite deformation in the tubular axis, resulting in the maximum length at the angle $\varphi^{*}$. At the angle $\varphi^{*}$, the change in the length of connected tubes is zero in linear deformation owing to the cancellation of the connected tubes.

Owing to the opposite behaviour of the non-rotated and rotated Miura-ori tubes, the length of the non-rotated tubes decreases as the angle φ increases. By contrast, the length of the rotated Miura-ori tubes increases, as shown in figure 2*c*. This opposite behaviour provides the maximum length for the connected tubes at $\varphi^{*}$ as indicated in figure 3, where the non-rotated and rotated Miura-ori tubes, defined by the parameters a=c=1 and $\alpha =55^{\circ }$, [36] are connected as a simple example. At the maximum length, the change in the length of the connected tubes is zero in the linear deformation because of the localization of the deformation in the connected tubes by the cancellation of kinematics. Moreover, the upper and lower bounds of the angle φ lock the motions of the non-rotated and rotated Miura-ori tubes, respectively.

### (b) Bar-and-hinge models for dispersion analysis

Elastic wave propagation is modelled using generalized Bloch wave frameworks with non-local interaction, based on bar-and-hinge models [42]. This is because bar-and-hinge models are widely used to capture the deformation caused by the kinematics of origami structures as the eigenmode (i.e. standing wave) of finite systems [57–59] and free wave propagation [42,60].

**Figure 4.**
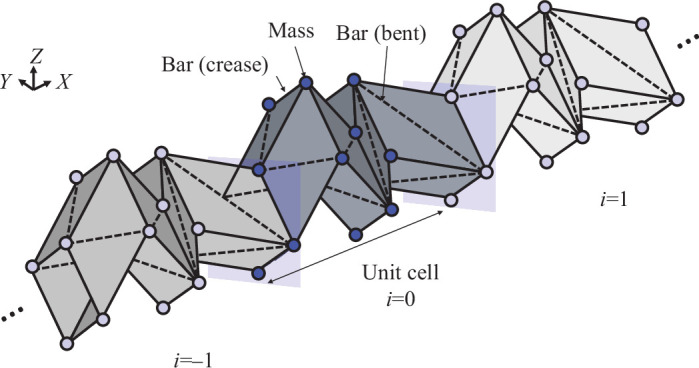
Schematic of bar-and-hinge models for dispersion analysis. The stiffness matrix and mass matrix are constructed using the N4B5 approach. Assuming the periodic systems along the X-axis, the unit cell with Bloch’s theorem calculates dispersion curves.

Using the bar-and-hinge models used in [42,61], the Miura-ori tubes were modelled using the N4B5 approach, where the parallelogram of Miura-ori was represented by four nodes and five bars, as illustrated in figure 4. The bar stiffness EA represents the shear and in-plane stiffness, where E is Young’s modulus and A is the cross-sectional area of the bar element. Furthermore, the rotational springs on the folding lines and diagonals of the panels contain rotational springs that represent the folding stiffness $K_{F}$ and bending stiffness $K_{B}$. The stiffness matrix of the N4B5 bar-and-hinge models were implemented based on MERLIN [62,63], where the stiffness matrix is expressed as


(2.8) $$K=C^{T}K_{S}C+J_{B}^{T}K_{B}J_{B}+J_{F}^{T}K_{F}J_{F},$$

where ${𝐊}_{S}$, ${𝐊}_{B}$ and ${𝐊}_{F}$ are diagonal matrices representing the stretch and shear stiffness of panels characterized by bar elements $K_{S}=EA/l$ (l is length of bar element), the bending of panels characterized by hinge elements on the diagonals of panels $K_{B}$ and the bending stiffness of creases characterized by rotational spring on the creases $K_{F}$. Furthermore, 𝐂, ${𝐉}_{B}$ and ${𝐉}_{F}$ are the compatibility matrices for the bar elements, and rotational springs for bending and folding, respectively.

Mass matrices were constructed by distributing the masses of the triangles surrounded by bars to the vertices of the triangles. Using the distributed mass $M_{1},...M_{N}$ for N nodes on the Miura-ori tubes, the mass matrix is expressed as a diagonal matrix:


(2.9) $$M=diag(M_{1},...,M_{N}).$$

For the mechanical systems defined by the stiffness and mass matrices 𝐊 and 𝐌, the generalized eigenvalue problem for each wave vector ${𝐤}_{m}$ can be obtained using the stiffness and mass matrices as follows:


(2.10) $$\begin{array}{ccc} & \tilde{𝐊}\tilde{𝐮}=\omega^{2}\tilde{𝐌}\tilde{𝐮}, & \end{array}$$

where the stiffness and mass matrices $\tilde{𝐊}$ and $\tilde{𝐌}$ are


(2.11) $$\tilde{K}=\sum_{j=-N}^{N}e^{-ik_{m}\cdot x_{i}}K_{0j},$$

(2.12) $$\tilde{M}=\sum_{j=-N}^{N}e^{-ik_{m}\cdot x_{i}}M_{0j},$$

where i is the imaginary unit, $x_{j}$ is the position of the j-th unit cell, $K_{0j}$ and $M_{0j}$ are the block matrices of the degrees of freedom of the j=0 and j-th unit cells, respectively and N is the number of unit cells of the non-local interaction. The geometry of the Miura-ori tube is defined by the parameters a=1 m, c=1 m and $\alpha =55^{\circ }$ [36]. The Young’s modulus E and cross-section area A, were set to $10^{5}$ Pa and 1 $m^{2}$, respectively. The folding and bending stiffnesses were defined by $K_{F}=1$ N⋅m/rad and $K_{B}=10$ N⋅m/rad, respectively [42]. The mass distribution was set by assuming a thickness of 1 m and a density of 1 $kg/m^{3}$. Additionally, the frequency in the dispersion curves was normalized to $\omega /\omega_{0}$ using $\omega_{0}=\sqrt{EA/aM_{p}}$, where $M_{p}$ is the mass of the parallelogram. The wave number k was normalized to kL/π, where L is the unit cell length defined in equation (2.7). A dispersion analysis was performed by solving eigenvalue problems defined by equation (2.10) for different wave vectors ${𝐤}_{m}$ (using MATLAB R2022b). The eigenvalue problem provides the set of wave number $k_{m}$ and wave modes as eigenvalues and eigenmodes, where the wave modes demonstrate the deformations for wave propagation.

To easily classify the wave modes of the Miura-ori tubes, the kinematic energy of wave modes was used [64]. The kinematic energy ratio κ of the wave mode in X-, Y- and Z- directions is defined as


(2.13) $$\kappa_{j}=\frac{\Gamma_{j}}{\sum_{j=1}^{3}\Gamma_{j}},$$

where $\Gamma_{j}$ is the kinematic energy of the j-th degree of freedom (j = 1, 2 and 3) and is expressed as


(2.14) $$\Gamma_{j}=\frac{1}{2}[(i\omega q_{j}{)}^{T}M_{00}(i\omega q_{j})],$$

where $q_{j}$ is components of j-th degree of freedom of the modal vector of the eigenvalue problem shown in equation (2.10); j=1,2 and 3 corresponded to X-, Y- and Z-directions.

## 3. Elastic wave propagation

### (a) Low-frequency wave mode by kinematics

**Figure 5.**
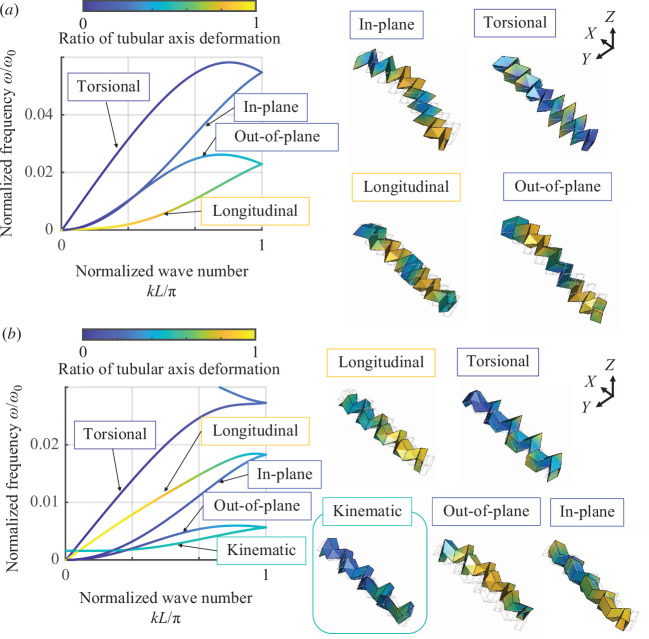
Dispersion curves of the Miura-ori tubes defined by the angle $\varphi =45^{\circ }$ are centralized in the range $\left[90^{\circ }-\alpha_{r}(=35^{\circ }),\alpha_{nr}(=55^{\circ })\right]$. The colour counters on dispersion curves indicate the ratio of tubular axis deformation of wave modes defined in equation (2.13). (*a*) Periodic non-rotated Miura-ori tubes. (*b*) Periodically connected non-rotated and rotated Miura-ori tubes. The connection generates additional wave modes characterized by the interaction of kinematics in non-rotated and rotated Miura-ori tubes.

The periodic structures of the non-rotated Miura-ori tubes and hybrid tubes connected by non-rotated and rotated Miura-ori tubes were examined to investigate the influence of the connection of Miura-ori tubes on the elastic wave propagation within the tubes, as shown in figure 5*a*,*b*. The colour counters on the dispersion curves indicate the ratio of tubular axis deformation of wave modes defined in equation (2.13). Representing the most symmetric geometry, a half-dihedral angle defined by the angle $\varphi =45^{\circ }$ located at the centre of the range $\left[90^{\circ }-\alpha_{r}(=35^{\circ }),\alpha_{nr}(=55^{\circ })\right]$ was used for the dispersion analysis. This condition extends to the maximum length of the hybrid tubes connected by the non-rotated and rotated Miura-ori tubes, as shown in figure 3.

Periodic non-rotated Miura-ori tubes have four wave modes, characterized by longitudinal and flexural waves (in-plane and out-of-plane) and torsional deformation at low frequencies, as shown in figure 5*a*. The vibration energy propagates in the tubes by different kinds of mechanisms such as longitudinal, torsional and bending. A longitudinal wave is a wave mode in which the vibration of tubes occurs along the parallel direction of the wave propagation (i.e. the direction in X-axis). The torsional wave is a wave mode whose deformation twists along the axis of wave propagation (i.e. deformation in the YZ-plane). Flexural waves are wave propagations with lateral deformations against the tubular axis. Considering that the structures have been surrounded by various kinds of excitations, band gaps for all wave modes are important to robustly prohibit the transportation of vibration energy. However, the acoustic branches of dispersion curves (i.e. the low-frequency region of dispersion curves such as classical acoustic waves) for these basic wave modes make it difficult to realize perfect low-frequency band gaps. Therefore, the complete low-frequency band gaps have been explored using quadruple-mode local resonance [65] and the chiral beam [66].

To realize complete band gaps, this study introduces localized wave modes by connecting the rotated Miura-ori tubes. Figure 5*b* shows the dispersion curves of the periodically connected non-rotated and $90^{\circ }$rotated Miura-ori tubes. This connection generates additional wave modes in $\omega /\omega_{0}$ from 0.002 to 0.005, which are not present in conventional Miura-ori tubes. The wave mode is illustrated as ‘kinematic’ in figure 5*b*. The interaction between the opposite kinematics of the non-rotated and rotated tubes generates additional wave modes that lead to localization of deformation in the unit cells owing to the cancellation of expansion and shrinkage. The localized wave mode does not couple with other wave modes, such as longitudinal, torsional and flexural waves at this folded state because of the ideal localization in the unit cell. This study controls the coupling of the localized wave mode and global elastic deformation by changing the folded state to form band gaps.

### (b) Effect of folded configuration on wave coupling

A dispersion analysis was performed for the connected Miura-ori tubes under different folded states by folding them to induce the wave coupling between the wave modes by kinematics and basic elastic deformation. In particular, the coupling of wave modes [67–69] has been discussed to understand the mechanisms behind tunable band gaps for elastic wave propagation in connected Miura-ori tubes.

Connecting the non-rotated and rotated Miura-ori tubes with the same geometry leads to symmetric deformation around the interfaces of the connection (i.e. relatively $90^{\circ }$-rotated along the tubular axis in each folded state), as shown in figure 3. Therefore, the fluctuation of the folding angles on one side from the state for the maximum length was investigated using angles $\varphi =45.5^{\circ },48^{\circ },51^{\circ }\;\;\text{and}\;\;54^{\circ }$ in $\varphi \in [35^{\circ },55^{\circ }]$.

**Figure 6.**
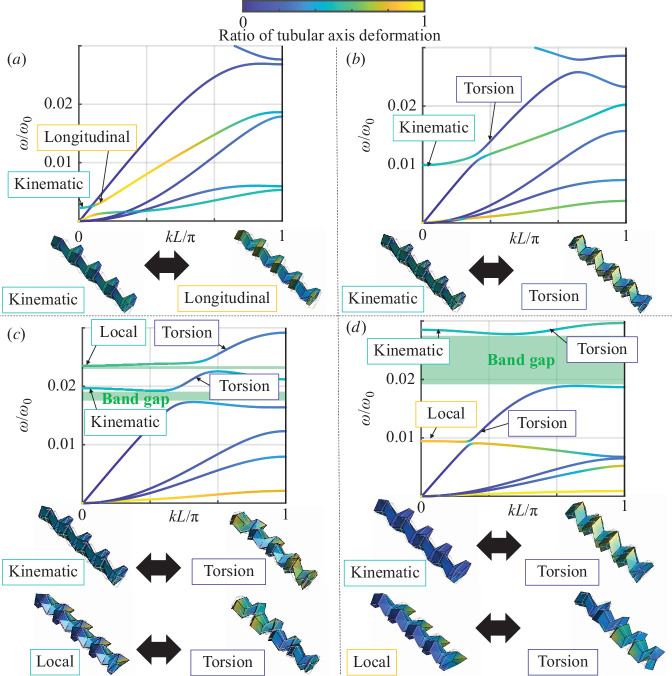
Dispersion curves of the waveguides connected between non-rotated and rotated Miura-ori tubes. (*a*), (*b*), (*c*) and (*d*) are results of the connecting by angles of interfaces $\varphi =45.5^{\circ },48^{\circ },51^{\circ }\;\text{and}\;54^{\circ }$.

The slight increase in angle φ from $45^{\circ }$ to $45.5^{\circ }$ brings the imperfection to localized kinematics. As a result, the mode is no longer isolated, and the coupling of the wave modes are caused as shown in figure 6*a*.

As the angle φ changes to $48^{\circ }$ as shown in figure 6*b*, the frequency of the kinematic wave mode increases, and the wave mode couples with the torsional wave modes. In this folded state, these wave modes are weakly coupled, as characterized by the veering of the dispersion curves [69]. Therefore, the coupling of the wave modes do not offer band gaps.

Further changes in the angle φ to $51^{\circ }$ are shown in figure 6*c*. Two wave modes at $\omega /\omega_{0}\neq 0$ and kL/π=0 can be observed, where the wave mode characterized by the local elastic deformation of the folded Miura-ori tubes occurs alongside the wave modes brought about by kinematics. These wave modes are strongly coupled to the torsional wave modes, resulting in two band gaps locking the wave propagation at approximately $\omega /\omega_{0}=0.02$ and $\omega /\omega_{0}=0.025$. Perfect low-frequency band gaps are formed by wave coupling in the acoustic branches of the torsional waves.

The rotated tube is folded further by setting the angle $\varphi =54^{\circ }$ near the lower bound of the angle range, as shown in figure 6*d*. Wide band gaps can be observed owing to the strong coupling of the wave modes caused by the kinematic and torsional modes. Additionally, as shown in figure 6*c*, the wave modes characterized by the local deformation of the folded tubes are shifted to approximately $\omega /\omega_{0}=0.01$, where the local deformation is dominated by tubular axial deformation and is weakly coupled to torsional waves.

These results suggest that while localized vibration caused by kinematics at $\varphi =\varphi^{*}$ is completely decoupled from other wave modes as shown in figure 5*b*, the fluctuation in φ changes the balance between the connected tubes with folding and unfolding motions, which leads to the coupling of the wave modes caused by kinematics to other wave modes. The tunable localized vibration caused by kinematics acts as a resonator and contributes to forming and tuning the low-frequency band gaps.

### (c) Effect of panel dimension on wave coupling

Non-rotated and rotated Miura-ori tubes with different panel geometries were used for the dispersion analysis to further explore the relationships between the dispersion curves and deformation of the connected Miura-ori tubes. The geometry of the rotated tube was defined by the parameters a=c=1 m and $\alpha =55^{\circ }$, which are the same as those described in §3*b*. By contrast, the geometrical parameter c of the non-rotated tube was changed to 1.2 m in this numerical example.

**Figure 7.**
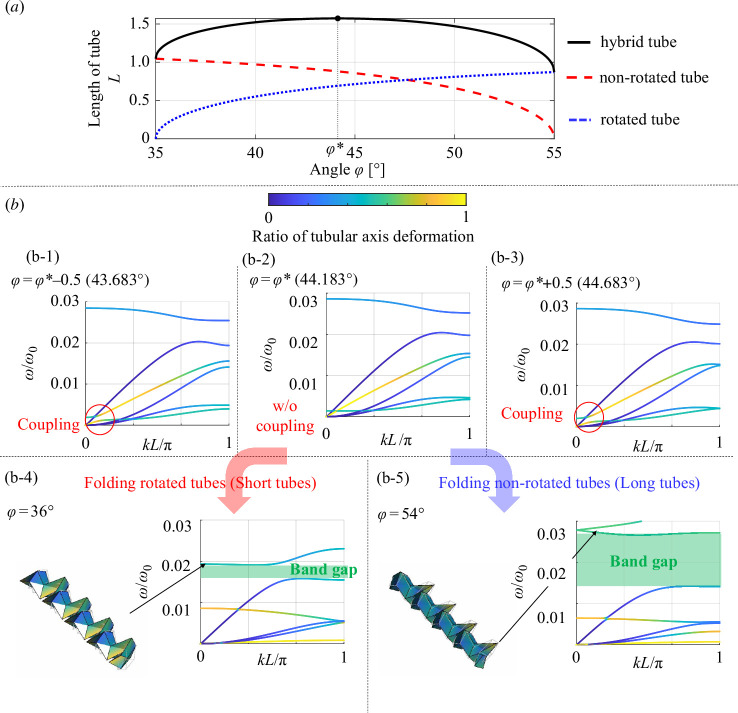
Dispersion curves obtained by connecting non-rotated and rotated Miura-ori tubes with different geometries. (*a*) Kinematics of the connected tubes with different geometries. (*b*) Dispersion curves of the connected tubes under the different folding states defined by $\varphi =36^{\circ },\varphi^{*}-0.5\;(=43.683^{\circ }),\varphi^{*}\;(=44.183^{\circ }),\varphi^{*}+0.5\;(=44.683^{\circ })$ and $54^{\circ }$. Folding of the Miura-ori tubes opens the band gap depending on the coupling strength of wave modes.

Considering that the length of the non-rotated and rotated tubes is different owing to the difference in the length of the parallelograms c, folding angles that cause the maximum length of the total length of the coupled tubes is different from the coupled tubes with the same value of c. Therefore, the asymmetry of the connected tubes shifts the angle $\varphi^{*}$ from $\varphi^{*}=45^{\circ }$ for connecting the same Miura-ori tubes to $\varphi^{*}=44.183^{\circ }$, as shown in figure 7*a*. As φ increases, the non-rotated Miura-ori tubes with c=1.2 m shrink, whereas the rotated Miura-ori tubes with c=1 m expand. Corresponding to the connection of Miura-ori tubes with the geometries shown in figure 5*b* , the connection of the non-rotated and rotated Miura-ori tubes with different geometries also provide uncoupled wave modes characterized by the kinematics of origami (i.e. cancellation of the opposite motion in the tubular axis) that can be observed at $\varphi =\varphi^{*}$ in figure 7(b-2)). Furthermore, the dispersion curves for $\varphi =\varphi^{*}-0.5$ and $\varphi =\varphi^{*}+0.5$ in figure 7(b-1),(b-3) indicate the coupling of wave modes between kinematic and longitudinal deformations as a result of slight fluctuations in the angle φ from $\varphi^{*}$.

The angle φ close to the upper and lower bounds leads to strong coupling of wave modes between the torsional wave modes and those caused by the kinematics observed in $\varphi =36^{\circ }$ and $\varphi =54^{\circ }$ in figure 7(b-4),(b-5), respectively. In the case of the coupling of Miura-ori tubes with different geometries, as shown in the dispersion curves of $\varphi =54^{\circ }$, the folding of the long Miura-ori tubes strongly couples the torsional modes compared with those of $\varphi =36^{\circ }$, thereby resulting in asymmetric band gaps between the angles φ close to the upper and lower bounds. These changes in the strength of the coupling of the wave modes offer various frequency ranges for band-gap formation.

### (d) Tunable wave coupling

**Figure 8.**
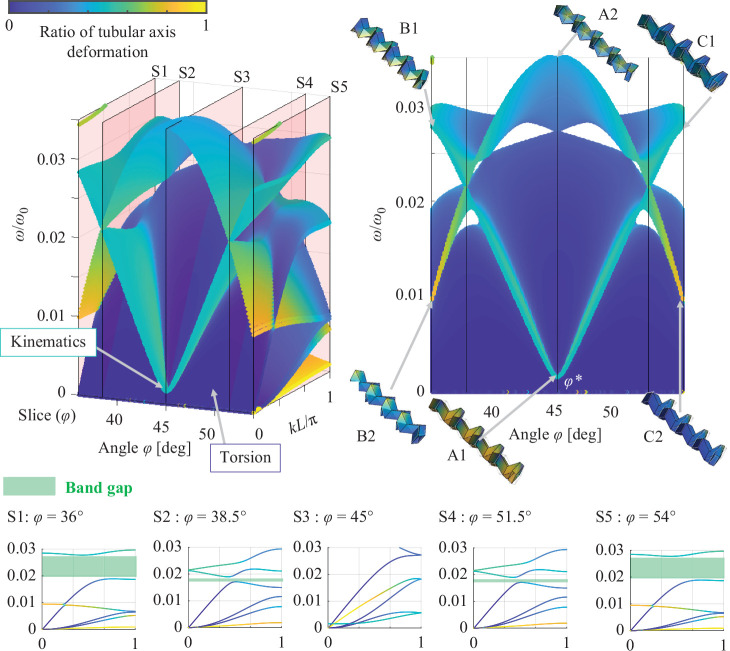
Shift of dispersion curves by the folding of coupled Miura-ori tubes with same length. Connection of Miura-ori tubes with same geometry defined by a=c=1 m and $\alpha =55^{\circ }$.

**Figure 9.**
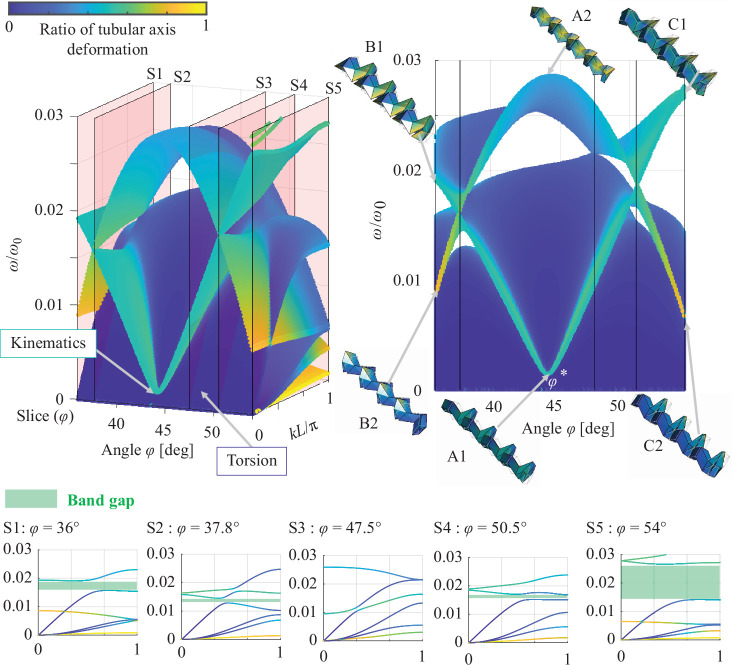
Shift of dispersion curves by folding of coupled Miura-ori tubes with different lengths. Connection of Miura-ori tubes with different geometries. The non-rotated tube is made longer than the rotated tube by changing the geometrical parameter c to 1.2 m.

The shift in the dispersion curves owing to the fluctuation of the angle φ for symmetric and asymmetric geometries discussed in §3*b*,*c* are shown in figures 8 and 9, which capture the change in the wave modes caused by the deformation of the connected Miura-ori tubes by folding. The surface illustrated in blue (i.e. a small ratio of tubular axis deformation) indicates changes in the dispersion curves characterized by the torsional wave modes. At a smaller wavenumber than the dispersion curves with torsional wave modes, the angle $\varphi^{*}$ provides wave modes by the cancellation of the non-rotated and rotated Miura-ori tubes at approximately kL/π=0 and low frequency $\omega /\omega_{0}$ in both symmetric (wave mode A1 in figure 8), and asymmetric geometries (wave mode A1 in figure 9), as discussed in §3*b* and §3*c*. Additionally, as a counterpart of these wave modes caused by ideal kinematics, the wave modes caused by elastic deformation of the panels occur at kL/π=0 and are higher than 0.03 in $\omega /\omega_{0}$ as shown in wave mode A2 in figures 8 and 9.

The fluctuation of the angle φ from $\varphi^{*}$ increases the frequency $\omega /\omega_{0}$ of the wave modes caused by the kinematics as a result of breaking the ideal cancellation of expansion and shrinking at angle $\varphi^{*}$. This results in the coupling of the wave modes, as shown in B1 and C1 in figures 8 and 9, respectively. Conversely, the counterpart of the wave modes at angle $\varphi^{*}$ shifts to the low-frequency wave mode by changing the shape of the wave mode to local deformations, such as B2 and C2 in figures 8 and 9, respectively.

Moreover, the coupling of wave modes, characterized by torsional deformation and kinematics, opens the band gap represented by the white areas in the side view of figure 8, which correspond to the band gaps shown in figure 6. Connecting the Miura-ori tubes with the same geometry provides symmetric band gaps along the angle $\varphi =45^{\circ }$ as shown in figure 8. To accurately capture the shifts of band gaps and the related dispersion curves, the dispersion curves are sliced from coloured surfaces as S1–S5 in figure 8. Thus, the shifts of band gaps caused by wave coupling (discussed in §3*b*) can be observed.

Conversely, coupling of the Miura-ori tubes with different geometries leads to the asymmetric formation of band gaps at $\varphi =44.183^{\circ }$ owing to variations in the coupling. The asymmetric changes in band gaps resulting from wave couplings are illustrated in slices of coloured surfaces labelled as S1–S5 in figure 9. Therefore, connecting non-rotated and rotated Miura-ori tubes with different geometries enables tuning of the band gap frequencies over a wider range.

## 4. Conclusion

This study proposes tunable elastic wave metamaterials based on the interaction of kinematics in non-rotated and rotated Miura-ori tubes. A dispersion analysis was performed to understand the behaviour of elastic waves in connected Miura-ori tubes to realize metamaterials. The findings of this study are as follows:

— Connecting non-rotated and rotated Miura-ori tubes along the tubular axis forms additional wave modes caused by the interaction between the kinematics of non-rotated and rotated Miura-ori tubes. The wave modes do not couple with the other wave modes because of the ideal cancellation of the deformation in the tubular axis at the state of the maximum length.
— Folding of the connected Miura-ori tubes leads to the coupling of the wave modes caused by the kinematics and the wave modes characterized by the global torsional deformation of Miura-ori tubes. The coupling of wave modes opens the complete low-frequency band gaps depending on the strength of wave-mode coupling.
— The connection of the Miura-ori tubes with different geometries provides asymmetric strength in coupling wave modes through the transformation of Miura-ori tubes via folding, resulting in band gaps in the various frequency ranges.

These findings suggest that periodically connected, rotated Miura-ori tubes have potential applications as switchable and tunable waveguides for vibration isolation in the desired frequency ranges via the transformation of the Miura-ori tubes by folding.

This study addresses hybrid Miura-ori tubes connected by limited geometries to understand the one-dimensional waveguides. Therefore, another type of connection for Miura-ori tubes has the potential for various kinematics to control wave propagation. Furthermore, two- or three-dimensional tessellation can control wave propagation at higher-order dimensions.

## Data Availability

This article has no additional data.

## References

[1] Dudte LH, Vouga E, Tachi T, Mahadevan L. 2016 Programming curvature using origami tessellations. Nat. Mater. **15**, 583–588. (10.1038/nmat4540) 26808459

[2] Narumi K, Koyama K, Suto K, Noma Y, Sato H, Tachi T, Sugimoto M, Igarashi T, Kawahara Y. 2023 Inkjet 4D print: self-folding tessellated origami objects by inkjet UV printing. ACM Trans. Graph. **42**, 1–13. (10.1145/3592409)

[3] Wang L, Saito K, Gotou Y, Okabe Y. 2019 Design and fabrication of aluminum honeycomb structures based on origami technology. Jnl. of Sandwich Struct. Mater. **21**, 1224–1242. (10.1177/1099636217714646)

[4] Naritomi D, Hosoya N, Ando G, Maeda S, Shigemune H. 2022 Creation of origami-inspired honeycomb structure using self-folding paper. Mater. Des. **223**, 111146. (10.1016/j.matdes.2022.111146)

[5] Zhang J, Karagiozova D, You Z, Chen Y, Lu G. 2019 Quasi-static large deformation compressive behaviour of origami-based metamaterials. Int. J. Mech. Sci. **153–154**, 194–207. (10.1016/j.ijmecsci.2019.01.044)

[6] Qiang W, Zhang J, Karagiozova D, Tran P, Lu G. 2021 Quasi-static energy absorption of miura-ori metamaterials. JOM **73**, 4177–4187. (10.1007/s11837-021-04939-w)

[7] Yuan L, Dai H, Song J, Ma J, Chen Y. 2020 The behavior of a functionally graded origami structure subjected to quasi-static compression. Mater. Des. **189**, 108494. (10.1016/j.matdes.2020.108494)

[8] Kuribayashi K, Tsuchiya K, You Z, Tomus D, Umemoto M, Ito T, Sasaki M. 2006 Self-deployable origami stent grafts as a biomedical application of ni-rich tini shape memory alloy foil. Mater. Sci. Eng. A **419**, 131–137. (10.1016/j.msea.2005.12.016)

[9] Fernandes R, Gracias DH. 2012 Self-folding polymeric containers for encapsulation and delivery of drugs. Adv. Drug Deliv. Rev. **64**, 1579–1589. (10.1016/j.addr.2012.02.012) 22425612 PMC3462897

[10] Li Y, Liu W, Deng Y, Hong W, Yu H. 2021 Miura-ori enabled stretchable circuit boards. npj Flex. Electron. **5**, 3. (10.1038/s41528-021-00099-8)

[11] Hou Y, Wang Y, Yu M, Wang Z, Yu H. 2020 Miura‐ori metastructure enhanced conductive elastomers. Adv. Mater. Technol. **5**, 2000249. (10.1002/admt.202000249)

[12] Miyashita S, Guitron S, Ludersdorfer M, Sung CR, Rus D. 2015 An untethered miniature origami robot that self-folds, walks, swims, and degrades. In IEEE International Conference on Robotics and Automation (ICRA), pp. 1490–1496. (10.1109/ICRA.2015.7139386)

[13] Shigemune H, Maeda S, Hara Y, Hosoya N, Hashimoto S. 2016 Origami robot: a self-folding paper robot with an electrothermal actuator created by printing. IEEE. ASME Trans. Mechatron. **21**, 2746–2754. (10.1109/TMECH.2016.2593912)

[14] Ze Q *et al*. 2022 Soft robotic origamic rawler. Sci. Adv. **8**. (10.1126/sciadv.abm7834) PMC8967224 35353556

[15] Ando K *et al*. 2020 Lightweight rigidly foldable canopy using composite materials. SN Appl. Sci. **2**, 1994. (10.1007/s42452-020-03846-0)

[16] Melancon D, Gorissen B, García-Mora CJ, Hoberman C, Bertoldi K. 2021 Multistable inflatable origami structures at the metre scale. Nature **592**, 545–550. (10.1038/s41586-021-03407-4) 33883736

[17] Gattas JM, You Z. 2016 Design and digital fabrication of folded sandwich structures. Autom. Constr. **63**, 79–87. (10.1016/j.autcon.2015.12.002)

[18] Georgakopoulos SV *et al*. 2021 Origami antennas. IEEE Open J. Antennas Propag. **2**, 1020–1043. (10.1109/OJAP.2021.3121102)

[19] Zirbel SA, Trease BP, Thomson MW, Lang RJ, Magleby SP, Howell LH. 2015 HanaFlex: a large solar array for space applications. In Micro- and nanotechnology sensors, systems, and applications VII (eds T George, AK Dutta, MS Islam), vol. 9467. Bellingham, WA: International Society for Optics and Photonics SPIE. (10.1117/12.2177730)

[20] Wilson L, Pellegrino S, Danner R. 2013 Origami Sunshield Concepts for Space Telescopes. In Structures, Structural Dynamics, and Materials Conference, Boston, Massachusetts, p. 10. Reston, Virginia: American Institute of Aeronautics and Astronautics. (10.2514/6.2013-1594). https://arc.aiaa.org/doi/book/10.2514/MSDM13.

[21] Miura K. 1975 New structural form of sandwich core. J. Aircr. **12**, 437–441. (10.2514/3.44468)

[22] Fischer S, Heimbs S, Kilchert S, Klaus M, Cluzel C. 2009 Sandwichstructures with folded core: manufacturing and mechanical behavior. In International Sampe Europe Conference. Paris.

[23] Xiang XM, Lu G, You Z. 2020 Energy absorption of origami inspired structures and materials. Thin-Walled Struct. **157**, 107130. (10.1016/j.tws.2020.107130)

[24] Ma J, You Z. 2014 Energy absorption of thin-walled square tubes with a prefolded origami pattern—part I: geometry and numerical simulation. J. Appl. Mech. **81**. (10.1115/1.4024405)

[25] Tachi T, Miura K. 2012 Rigid-foldable cylinders and cells. J. Int. Assoc. Shell. Spatial. Struct. **53**, 217–226.

[26] Yasuda H, Yein T, Tachi T, Miura K, Taya M. 2013 Folding behaviour of tachi-miura polyhedron bellows. Proc. R. Soc. A **469**, 20130351. (10.1098/rspa.2013.0351) 24204186 PMC3780822

[27] Schenk M, Guest SD. 2013 Geometry of miura-folded metamaterials. Proc. Natl Acad. Sci. USA **110**, 3276–3281. (10.1073/pnas.1217998110) 23401549 PMC3587190

[28] Yasuda H, Yang J. 2015 Reentrant origami-based metamaterials with negative poisson’s ratio and bistability. Phys. Rev. Lett. **114**, 185502. (10.1103/PhysRevLett.114.185502) 26001009

[29] Xiang X, Qiang W, Hou B, Tran P, Lu G. 2020 Quasi-static and dynamic mechanical properties of miura-ori metamaterials. Thin-Walled Struct. **157**, 106993. (10.1016/j.tws.2020.106993)

[30] Li S, Wang KW. 2015 Fluidic origami with embedded pressure dependent multi-stability: a plant inspired innovation. J. R. Soc. Interface **12**, 20150639. (10.1098/rsif.2015.0639) 26400197 PMC4614500

[31] Li S, Fang H, Wang KW. 2016 Recoverable and programmable collapse from folding pressurized origami cellular solids. Phys. Rev. Lett. **117**, 114301. 10.1103/PhysRevLett.117.114301. (10.1103/PhysRevLett.117.114301) 27661691

[32] Yasuda H, Gopalarethinam B, Kunimine T, Tachi T, Yang J. 2019 Origami‐Based cellular structures with in situ transition between collapsible and load‐bearing configurations. Adv. Eng. Mater. **21**, 1900562. 10.1002/adem.201900562. (10.1002/adem.201970037)

[33] Tomita S, Shimanuki K, Nishigaki H, Oyama S, Sasagawa T, Murai D, Umemoto K. 2023 Origami-inspired metamaterials with switchable energy absorption based on bifurcated motions of a tachi-miura polyhedron. Mater. Des. **225**, 111497. (10.1016/j.matdes.2022.111497)

[34] Tomita S *et al*. 2023 Transition of deformation modes from bending to auxetic compression in origami-based metamaterials for head protection from impact. Sci. Rep. **13**, 12221. (10.1038/s41598-023-39200-8) 37500726 PMC10374913

[35] Tomita S, Shimanuki K, Umemoto K. 2024 Control of buckling behavior in origami-based auxetic structures by functionally graded thickness. J. Appl. Phys. **135**. (10.1063/5.0194238)

[36] Filipov ET, Tachi T, Paulino GH. 2015 Origami tubes assembled into stiff, yet reconfigurable structures and metamaterials. Proc. Natl Acad. Sci. USA **112**, 12321–12326. (10.1073/pnas.1509465112) 26351693 PMC4603468

[37] Tomita S, Shimanuki K, Umemoto K, Kawamoto A, Nomura T, Tachi T. 2023 Coupled thick-panel origami tubes along creases for stiff deployable structures. In Proceedings of IASS Annual Symposia 2023, vol. 19, pp. 1–9,

[38] Yasuda H, Chong C, Charalampidis EG, Kevrekidis PG, Yang J. 2016 Formation of rarefaction waves in origami-based metamaterials. Phys. Rev. E **93**, 043004. (10.1103/PhysRevE.93.043004) 27176382

[39] Yasuda H, Miyazawa Y, Charalampidis EG, Chong C, Kevrekidis PG, Yang J. 2019 Origami-based impact mitigation via rarefaction solitary wave creation. Sci. Adv. **5**, eaau2835. (10.1126/sciadv.aau2835) 31139744 PMC6534386

[40] Xu ZL, Wang DF, Tachi T, Chuang KC. 2022 An origami longitudinal–torsional wave converter. Extreme Mech. Lett. **51**, 101570. (10.1016/j.eml.2021.101570)

[41] Wang DF, Wang YQ, Qian ZH, Tachi T, Chuang KC. 2021 A graded miura-ori phononic crystals lens. Phys. Lett. A **418**, 127701. (10.1016/j.physleta.2021.127701)

[42] Pratapa PP, Suryanarayana P, Paulino GH. 2018 Bloch wave framework for structures with nonlocal interactions: application to the design of origami acoustic metamaterials. J. Mech. Phys. Solids **118**, 115–132. (10.1016/j.jmps.2018.05.012)

[43] Jiang T, Li C, Han Q. 2022 Tunable polarization bandgaps and elastic wave transmission in anisotropic origami metamaterials. Waves in Rand. Complex Med. 1–23. (10.1080/17455030.2022.2091810)

[44] Nanda A, Karami MA. 2018 Tunable bandgaps in a deployable metamaterial. J. Sound Vib. **424**, 120–136. (10.1016/j.jsv.2018.03.015)

[45] Jiang T, Han Q, Li C. 2023 Topologically tunable local-resonant origami metamaterials for wave transmission and impact mitigation. J. Sound Vib. **548**, 117548. (10.1016/j.jsv.2022.117548)

[46] Yasuda H, Yang J. 2017 Tunable frequency band structure of origami-based mechanical metamaterials. J. Int. Assoc. Shell Spatial Struct. **58**, 287–294. (10.20898/j.iass.2017.194.905)

[47] Zhang M, Yang J, Zhu R. 2021 Origami-based bistable metastructures for low-frequency vibration control. J. Appl. Mech. **88**. (10.1115/1.4049953)

[48] Xu ZL, Wang YQ, Zhu R, Chuang KC. 2021 Torsional bandgap switching in metamaterials with compression–torsion interacted origami resonators. J. Appl. Phys. **130**. (10.1063/5.0056179)

[49] Miyazawa Y, Chen CW, Chaunsali R, Gormley TS, Yin G, Theocharis G, Yang J. 2022 Topological state transfer in kresling origami. Commun. Mater. **3**. (10.1038/s43246-022-00280-0)

[50] Zhao P, Zhang K, Deng Z. 2022 Origami-inspired lattice for the broadband vibration attenuation by symplectic method. Ext. Mech. Lett. **54**, 101771. (10.1016/j.eml.2022.101771)

[51] Zhang X, Huang X, Lu G. 2023 Tunable bandgaps and acoustic characteristics of perforated miura-ori phononic structures. Int. J. Mech. Sci. **253**, 108389. (10.1016/j.ijmecsci.2023.108389)

[52] Babaee S, Overvelde JTB, Chen ER, Tournat V, Bertoldi K. 2016 Reconfigurable origami-inspired acoustic waveguides. Sci. Adv. **2**, e1601019. (10.1126/sciadv.1601019) 28138527 PMC5262461

[53] Thota M, Li S, Wang KW. 2017 Lattice reconfiguration and phononic band-gap adaptation via origami folding. Phys. Rev. B **95**, 064307. (10.1103/PhysRevB.95.064307)

[54] Thota M, Wang KW. 2018 Tunable waveguiding in origami phononic structures. J. Sound Vib. **430**, 93–100. (10.1016/j.jsv.2018.05.031)

[55] Fang H, Li S, Thota M, Wang KW. 2019 Origami lattices and folding-induced lattice transformations. Phys. Rev. Research **1**, 023010. (10.1103/PhysRevResearch.1.023010)

[56] Liu KT, Paulino GH. 2024 Geometric mechanics of hybrid origami assemblies combining developable and non-developable patterns. Proc.R. Soc. A **480**, 20230716. (10.1098/rspa.2023.0716)

[57] Cheung KC, Tachi T, Calisch S, Miura K. 2014 Origami interleaved tube cellular materials. Smart Mater. Struct. **23**, 094012. (10.1088/0964-1726/23/9/094012)

[58] Filipov ET, Paulino GH, Tachi T. 2016 Origami tubes with reconfigurable polygonal cross-sections. Proc. R. Soc. A **472**, 20150607. (10.1098/rspa.2015.0607) 26997894 PMC4786039

[59] Filipov ET, Liu K, Tachi T, Schenk M, Paulino GH. 2017 Bar and hinge models for scalable analysis of origami. Int. J. Solids Struct. **124**, 26–45. (10.1016/j.ijsolstr.2017.05.028)

[60] Oudghiri-Idrissi O, Guzina BB. 2022 Effective linear wave motion in periodic origami structures. Comput. Methods Appl. Mech. Eng. **399**, 115386. (10.1016/j.cma.2022.115386)

[61] Liu K, Paulino GH. 2017 Nonlinear mechanics of non-rigid origami: an efficient computational approach. Proc.R. Soc. A Math. Phys. Eng. Sci. **473**, 20170348. (10.1098/rspa.2017.0348) PMC5666233 29118663

[62] Liu K, Paulino GH. MERLIN: a MATLAB implementation to capture highly nonlinear behavior of non-rigid origami. In Proceedings of IASS Annual Symposia, vol. 13, pp. 1–10,

[63] Liu K, Paulino.2018 G. Highly efficient nonlinear structural analysis of origami assemblages using the MERLIN2 software. Origami **7**, 1167–1182.

[64] Hong J, He X, Zhang D, Zhang B, Ma Y. 2018 Vibration isolation design for periodically stiffened shells by the wave finite element method. J. Sound Vib. **419**, 90–102. (10.1016/j.jsv.2017.12.035)

[65] Fujita K, Tomoda M, Wright OB, Matsuda O. 2019 Perfect acoustic bandgap metabeam based on a quadruple-mode resonator array. Appl. Phys. Lett. **115**. (10.1063/1.5117283)

[66] Park J, Lee D, Jang Y, Lee A, Rho J. 2022 Chiral trabeated metabeam for low-frequency multimode wave mitigation via dual-bandgap mechanism. Commun. Phys. **5**, 194. (10.1038/s42005-022-00974-4)

[67] Yaman Y. 1997 Vibrations of open-section channels: a coupled flexural and torsional wave analysis. J. Sound Vib. **204**, 131–158. (10.1006/jsvi.1997.0933)

[68] Bhaskar A. 2003 Waveguide modes in elastic rods. Proc. R. Soc. Lond. A.Math, Phys Eng Sci **459**, 175–194. (10.1098/rspa.2002.1013)

[69] Mace BR, Manconi E. 2012 Wave motion and dispersion phenomena: veering, locking and strong coupling effects. J. Acoust. Soc. Am. **131**, 1015–1028. (10.1121/1.3672647) 22352477

